# Tuberculosis disease burden and attributable risk factors in Nigeria, 1990–2016

**DOI:** 10.1186/s41182-018-0114-9

**Published:** 2018-09-25

**Authors:** Felix Akpojene Ogbo, Pascal Ogeleka, Anselm Okoro, Bolajoko O. Olusanya, Jacob Olusanya, Ifegwu K. Ifegwu, Akorede O. Awosemo, John Eastwood, Andrew Page

**Affiliations:** 1 Translational Health Research Institute, School of Medicine, Western Sydney University, Penrith, New South Wales Australia; 2 Prescot Specialist Medical Centre, Welfare Quarters, Makurdi, Benue State Nigeria; 3 Society for Family Health, Justice Ifeyinwa Nzeako House, 8 Port Harcourt Crescent Area 11, Garki, Abuja, Nigeria; 4 Centre for Healthy Start Initiative, 286A Corporation Drive, Dolphin Estate, Ikoyi, Lagos, Nigeria; 5 Ingham Institute for Applied Medical Research, 1 Campbell Street, Liverpool, New South Wales 2170 Australia; 6 School of Women’s and Children’s Health, The University of New South Wales, Kensington, Sydney, New South Wales 2052 Australia; 7 School of Public Health, The University of Sydney, Sydney, New South Wales 2006 Australia; 8 School of Public Health, Griffith University, Queensland, Gold Coast, 4222 Australia; 9 Department of Community Paediatrics, Sydney Local Health District, Croydon Community Health Centre, 24 Liverpool Rd, Croydon, New South Wales 2132 Australia

**Keywords:** Tuberculosis, Burden, Nigeria, Mortality, Global burden of disease

## Abstract

**Background:**

According to the World Health Organization, Nigeria is one of the countries with a high burden of tuberculosis (TB) worldwide. Improving the burden of TB among HIV-negative people would require comprehensive and up-to-date data to inform targeted policy actions in Nigeria. The study aimed to describe the incidence, prevalence, mortality, disability-adjusted life years (DALYs) and risk factors of tuberculosis in Nigeria between 1990 and 2016.

**Methods:**

This study used the most recent data from the global burden of disease study 2016. TB deaths were estimated using the Cause of Death Ensemble model, while TB incidence, prevalence and DALYs, as well as years of life lost and years of life lived with disability were calculated in the DisMod-MR 2.1, a Bayesian meta-regression tool. Using a comparative risk assessment approach, TB burden attributable to risk factors was estimated in a spatial-temporal Gaussian Process Regression tool.

**Results:**

In 2016, the prevalence of TB among HIV-negative people was 27% (95% uncertainty interval [95% UI] 23–31%) in Nigeria. TB incidence rate (new and relapse cases) was 158 per 100,000 people (95% UI; 128-193), while the total number of TB mortality was 39,933 deaths (95% UI; 30,488-55,039) in 2016. Between 2000 and 2016, the age-standardised prevalence and incidence rates of TB-HIV negative decreased by 20.0 and 87.6%, respectively. The age-standardised mortality rate also dropped by 191.6% over the same period. DALYs due to TB among HIV-negative Nigerians was high but varied across the age groups. Of the risk factors studied, alcohol use accounted for the highest number of TB deaths and DALYs, followed by diabetes and smoking in 2016.

**Conclusion:**

The study shows an improving trend in TB disease burden among HIV-negative individuals in Nigeria from 1990 to 2016. Despite this progress, this study suggests that additional efforts are still needed to ensure that Nigeria is not left behind in the current global strategy to end TB disease. Reducing TB disease burden in the country will require a multipronged approach that includes increased funding, health system strengthening and improved TB surveillance, as well as preventive efforts for alcohol use, smoking and diabetes.

**Electronic supplementary material:**

The online version of this article (10.1186/s41182-018-0114-9) contains supplementary material, which is available to authorized users.

## Background

Tuberculosis (TB) remains a significant public health issue in low-income and middle-income countries and is the leading cause of deaths as a single infectious disease, ranking above human immunodeficiency virus and acquired immune deficiency syndrome (HIV/AIDS) [1]. The World Health Organization’s (WHO) Global Tuberculosis Report 2017 reported 6.3 million new cases of TB among HIV-negative people in 2016 [1], compared to 6.1 million in 2015 [2]. Similarly, the Global Burden of Diseases, Injuries and Risk Factors (GBD) Study 2016 estimated 9.0 million TB-HIV-negative incident cases (new and relapse cases) compared to 8.8 million in 2015 [3]. These reports highlighted the considerable burden of TB globally. For example, the WHO African region accounted for 25% of the total number of incident cases (i.e., TB-HIV-negative and TB-HIV infection) globally, where Nigeria accounted for 8% or 407 cases per 100,000 population in 2016 [1], up from 322 cases per 100,000 population in 2015 [2]. These estimates may be lower than the actual number of TB cases in Nigeria because only less than a quarter of TB cases (15%) were notified in 2015 [2].

In the past two decades, the WHO has listed Nigeria as one of the countries with a high burden of TB in order to stimulate targeted interventions and advocacy for funding and policies to improve TB control [4]. This initiative has led to focused and practical actions for TB control worldwide [1]. Recently, the Nigeria National TB Control Programme and its donor partners have commenced the scale-up of availability and accessibility to improved methods for TB diagnosis and effective treatment regimen [5, 6]. While those efforts are needed and well deserved in Nigeria, there are limited pragmatic policy actions to tackle emerging risk factors for TB at the population level, including diabetes [7, 8], alcohol intake [8–11] and tobacco smoking [8, 12]. Country-specific epidemiologic studies which investigate trends in TB disease burden and the attributable risk factors for TB would be useful for public health experts and policy-makers to strengthen TB control and preventive efforts.

Evidence shows that TB mortality among HIV-negative people has declined in many developing countries (including Nigeria); but that TB incidence has remained unchanged in many communities [1, 3]. To ensure a continued reduction in TB disease burden in Nigeria, it is essential to understand not only the trends in TB burden but also the extent to which risk factors contribute to TB disease burden to inform targeted and high-priority TB programmes. We have provided a detailed exposition of TB disease burden in Nigeria from the GBD findings because this is not practicable in the GBD capstone publications due to the huge size and scope of the study, which have also led to further characterisation of the results for other health focus areas and locations [3, 13–16]. Additionally, by distilling the findings for TB burden in Nigeria, we aim to increase awareness and understanding of TB estimates for clinicians, national, and international health experts for TB prevention and control programmes, especially that Nigeria is the largest recipient of developmental assistance for health in Sub-Saharan Africa [17]. The present study aimed to highlight the incidence, prevalence, deaths, disability-adjusted life years (DALYs) and risk factors for tuberculosis in Nigeria from 1990 to 2016 using data from the GBD Study 2016.

## Methods

### Overview of data sources

The GBD study is a systematic and scientific effort that provides comparable estimates of incidence, prevalence, the cause of death and health loss, and risk factors for diseases and injuries by age, sex, year, location, and over time. In the past two decades, the GBD study has been quantifying health loss from diseases and injuries to inform health programmes and policy decision-making worldwide [18, 19]. The GBD 2016 complied with the Guidelines for Accurate and Transparent Health Estimates Reporting (GATHER) statement, a global agreement that ensures transparency, accurate reporting, interpretation and use of health estimates [20].

For this study, the complete information on data sources, the conceptual framework, and the analytical strategy for the calculation of TB incidence, prevalence, mortality, DALYs and attributable risk factors in Nigeria has been described elsewhere [3, 21–25]. Data used for the TB estimation in Nigeria have been extracted from the Global Health Exchange website (GHDx, http://ghdx.healthdata.org/gbd-2016/data-input-sources). GHDx provides researchers and policy-makers access to the most recent GBD input sources and results, and also creates opportunities for discussing population health based on the best available data, as well as acknowledgment of data owners’ contributions [26].

### Case definition

TB is an infectious disease caused by the bacterium *Mycobacterium tuberculosis*, an acid-fast bacillus that is spread mainly via the respiratory pathway. The GBD study provides estimates for all forms of TB, including pulmonary and extrapulmonary TB using the International Classification of Diseases (ICD-10) codes [27]. In this study, we have reported estimates for TB (drug-susceptible TB, extensively drug resistance TB, latent TB infection and multidrug-resistant TB, MDR-TB) among HIV-negative people in Nigeria. Information on TB-HIV is provided elsewhere [3, 28].

### Overview of the estimation of incidence, prevalence, mortality, disability-adjusted life years and risk factors for tuberculosis

TB mortality was modelled in the GBD Cause of Death Ensemble model (CODEm), a Bayesian, hierarchical, ensemble modelling tool, which has been used to estimate cause-specific mortality for a range of diseases and injuries globally [21, 29]. CODEm modelling strategy used data from the WHO Global Project on Anti-Tuberculosis Drug Resistance Surveillance data (1988–2015) and community-based surveillance data for Nigeria and applied different functional forms (mixed-effects models and spatiotemporal Gaussian process regression models) to mortality rates with varying combinations of predictive models [21].

TB incidence was estimated based on age-specific and sex-specific notification data from the WHO and was defined as new and relapse cases diagnosed within a given calendar year [25]. Categorised notification data (i.e. new pulmonary smear-positive, new pulmonary smear-negative, new extrapulmonary and relapse) were combined to represent all forms of TB [3]. The GBD study estimated point prevalence of TB, defined as the people in the population who at any point within a calendar year with active TB [25].

DALYs are a summary metric of disease or injuries, defined as the number of years lost due to ill-health, disability or premature death, and were computed as the sum of years of life lost (YLLs) and years lived with disability (YLDs) for each year and age in Nigeria [24]. YLLs were calculated by multiplying TB deaths by normative standard life birth (86.9 years), measured as the lowest observed death rates for each 5-year age group in populations higher than five million [30]. In the estimation of YLDs, TB epidemiologic data from the WHO and the Nigeria National Tuberculosis Prevalence Survey 2012 were multiplied by a TB-specific disability weight. The disability weight was obtained from population-based surveys, where respondents rated their health status, from ‘perfect health’ to ‘death’ to quantify the severity of the health loss due to a given disease or injury [24].

TB mortality and DALYs attributable to risk factors were computed as the proportion of deaths and DALYs that could be attributed to risk factors (alcohol use, diabetes and tobacco smoking) as a counterfactual relative to the theoretical minimum level of exposure had the population not been exposed to the given risk factor previously. Based on the available evidence on the causal relationship between risk factors and TB, GBD 2016 estimated the attributable burden of diabetes, alcohol use and tobacco smoking for TB in Nigeria using the comparative risk assessment (CRA) strategy developed by Murray and Lopez [31]. Estimates of the attributable number of deaths or DALYs were calculated by multiplying the number of deaths, or DALYs for the outcome by the population attributable fraction (PAF) for the risk-outcome pair for a given age and year in Nigeria [3].

The analyses were conducted in DisMod-MR 2.1, the GBD meta-regression tool that adjusts for variations in epidemiologic data sources and other parameters, including model predictions, as well as propagates uncertainty around the estimates. DisMod-MR 2.1 also estimated 95% corresponding uncertainty intervals for TB incidence, prevalence, deaths and DALYs. A full description of the analytical strategy for the estimation of TB epidemiology in Nigeria is provided in respective GBD study publications [21–25].

## Results

### Levels and trends of tuberculosis prevalence, incidence, mortality and DALYs

**Fig. 1:**
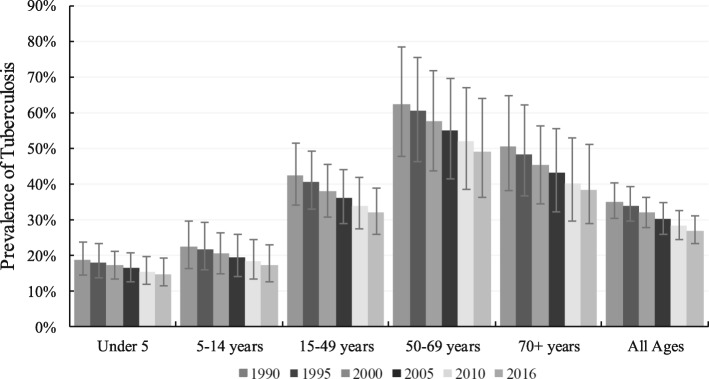
Prevalence of tuberculosis in Nigeria by age, 1990–2016

In 2016, age-standardised prevalence rate of TB among HIV-negative people was 31,643.5 per 100,000 population (95% uncertainty interval [95% UI] 27,316-36,249) (Table 1), while the absolute prevalence was 27% (95% UI; 23–31%), highest in people aged 50–69 years and lowest in children under 5 years (Fig. 1). Absolute TB incidence rate (new and relapse cases) was 158 per 100,000 people (95% UI; 128-193) (Table 2).

**Table 1:** Age-standardised cases of tuberculosis, drug-susceptible tuberculosis, multidrug-resistant tuberculosis and extensively drug-resistant tuberculosis among HIV-negative individuals in Nigeria, 2000–2016

	Prevalence			Incidence		
	2000	2016	% change, 2000–2016	2000	2016	% change, 2000–2016
	Rate/100,000 (95% UI)	Rate/100,000 (95% UI)		Rate/100,000 (95% UI)	Rate/100,000 (95% UI)	
Tuberculosis	37,964.1 (32,963.3–43,057.0)	31,643.5 (27,316.3–36,249.3)	−20.0%	373.9 (300.2–455.4)	199.2 (162.0–238.5)	−87.6%
Drug-susceptible tuberculosis	269.9 (217.2–329.6)	147.3 (119.4–180.2)	−83.3%	362.8 (292.3–442.1)	192.9 (157.3–231.8)	−88.0%
Multidrug-resistant tuberculosis	8.2 (2.7–18.5)	4.8 (2.0–9.5)	−72.5%	11.1 (3.8–25.2)	6.3 (2.7–12.8)	−76.6%
Latent tuberculosis infection	37,685.9 (32,701.1–42,774.3)	31,491.4 (27,161.1–36,074.3)	−19.7%	–	–	–
	Deaths			DALYs		
Tuberculosis	131.3 (101.7–177.9)	45.0 (35.2–59.3)	−191.6%	3524.8 (2696.8–4867.8)	1159.3 (897.4–1557.6)	−204.1%
Drug-susceptible tuberculosis	120.0 (92.0–163.4)	40.9 (31.6–54.2)	−193.2%	3228.3 (2414.1–4569.2)	1056.5 (808.5–1427.6)	−205.6%
Multidrug-resistant tuberculosis	11.3 (3.8–25.0)	4.0 (1.7–7.9)	−179.1%	295.7 (98.2–640.7)	101.1 (43.0–199.7)	− 192.5%
Extensively drug-resistant tuberculosis	–	–	–	0.9 (0.3–1.9)	1.7 (0.7–3.4)	48.1%

- indicate less than one per 100,000 population

**Table 2:** Incidence rate of tuberculosis (with 95% uncertainty interval, UI) by age in Nigeria, 1990–2016 (per 100,000 population)

Age	1990	1995	2000	2005	2010	2016	% change (1990–2016)
	*N* (95% UI)	*N* (95% UI)	*N* (95% UI)	*N* (95% UI)	*N* (95% UI)	*N* (95% UI)	
Under 5 years	218 (153–306)	226 (156–323)	220 (150–317)	179 (122–264)	136 (91–199)	102 (71–148)	− 53.3
5–14 years	68 (43–100)	71 (45–106)	78 (48–117)	77 (47–117)	65 (38–102)	56 (34–85)	− 16.9
15–49 years	356 (273–461)	377 (273–497)	358 (254–487)	290 (201–400)	228 (161–312)	210 (155–274)	− 41.1
50–69 years	536 (399–699)	558 (398–742)	557 (377–773)	474 (321–669)	354 (235–506)	276 (182–396)	− 48.6
70+ years	799 (591–1045)	807 (601–1073)	759 (543–1007)	607 (428–821)	425 (304–574)	342 (244–464)	− 57.2
All ages	277 (233–329)	292 (240–356)	285 (228–352)	236 (188–293)	182 (146–231)	158 (128–193)	− 42.9

**Fig. 2:**
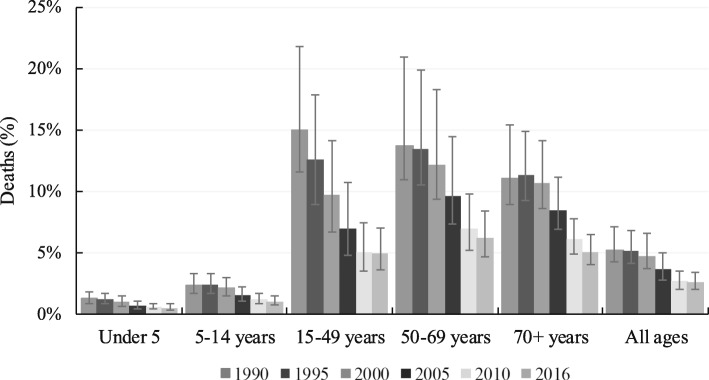
Prevalence of deaths from tuberculosis in Nigeria by age, 1990–2016

In the same year, the total number of TB mortality was 39,933 deaths (95% UI; 30,488-55,039), highest in people aged 15–49 years (13,916, 95% UI; 9311-20,530) but lowest in those aged between 5 and 14 years (875, 95% UI; 600-1,211) (Table 3). A similar pattern in the prevalence of TB mortality was observed (Fig. 2). Between 2000 and 2016, the age-standardised prevalence and incidence rates of TB-HIV negative decreased by 20.0 and 87.6%, respectively. The age-standardised mortality rate also dropped by 191.6% over the same period. Drug-susceptible TB was the most common variant, followed by multidrug- resistance TB in 2016 (Table 1).

**Table 3:** Number of deaths from tuberculosis (with 95% uncertainty interval, UI) by age in Nigeria, 1990–2016

Age	1990	1995	2000	2005	2010	2016	% change (1990–2016)
	*N* (95% UI)	*N* (95% UI)	*N* (95% UI)	*N* (95% UI)	*N* (95% UI)	*N* (95% UI)	
Under 5 years	9313 (5939–13,651)	9557 (6136–14,356)	8577 (5421–13,213)	5868 (3733–8747)	3519 (2148–5743)	4720 (3030–7196)	− 49.3
5–14 years	1382 (964–1926)	1517 (1055–2095)	1447 (981–1975)	1113 (756–1572)	746 (503–1060)	875 (600–1211)	− 36.7
15–49 years	21,542 (16,162–31,474)	24,437 (17,214–34,397)	25,107 (16,951–37,381)	20,300 (13,190–30,585)	12,187 (8543–17,420)	13,916 (9311–20,530)	− 35.4
50–69 years	18,723 (14,227–27,847)	21,405 (15,544–31,463)	22,178 (15,703–32,747)	17,871 (12,701–26,285)	10,573 (7688–1,4845)	12,357 (8797–17,817)	− 34
70+ years	11,553 (9091–15,539)	13,613 (10,740–17,887)	14,034 (10,930–18,725)	11,274 (8711–14,819)	7377 (5655–9722)	8065 (6129–10,550)	− 30.2
All ages	62,513 (50,969–85,245)	70,530 (54664–94,278)	71,343 (54,497–98,715)	56,427 (42,423–77,678)	34,403 (26,533–46,550)	39,933 (30,488–55,039	− 36.1

In Nigeria, the burden of TB among HIV-negative people was highest in those aged 15–49 years (660,942 DALYs [477,430-921,111]), followed by people aged 50–69 years (312,294, 95% UI; 227,215-440,406) (Table 4).

**Table 4:** Numbers of disability-adjusted life years (with 95% uncertainty interval, UI) due to tuberculosis in Nigeria, 1990–2016

Age	1990	1995	2000	2005	2010	2016	% change (1990–2016)
	*N* (95% UI)	*N* (95% UI)	*N* (95% UI)	*N* (95% UI)	*N* (95% UI)	*N* (95% UI)	
Under 5 years	796,326 (509,536–1,167,425)	818,094 (526,989–1,225,994)	735,514 (466,850–1,131,697)	504,739 (325,725–750,223)	406,505 (263,324–617,181)	303,628 (186,059–490,830)	− 61.9
5–14 years	109,450 (77,440–151,691)	120,172 (84,493–165,691)	115,521 (78,775–155,780)	90,261 (62,470–125,194)	71,786 (50,534–99,023)	61,720 (43,117–86,028)	− 43.6
15–49 years	1,144,635 (872,156–1,633,936)	1,308,897 (930,238–1,818,531)	1,349,558 (922,957–1,973,510)	1,089,776 (723,744–1,636,032)	755,490 (520,518–1,080,864)	660,942 (477,430–921,111)	− 42.3
50–69 years	541,198 (411,353–813,917)	619,595 (449,209–901,830)	643,310 (455,098–948,833)	519,960 (372,897–763,299)	363,631 (259,050–524,544)	312,294 (227,215–440,406)	− 42.3
70+ years	162,936 (127,597–221,855)	191,308 (149,138–256,179)	196,198 (150,260–266,630)	156,696 (119,232–207,194)	111,538 (84,317–148,261)	101,646 (77,262–134,947)	− 37.6
All ages	2,754,545 (2,222,630–3,625,684)	3,058,066 (2,396,218–3,929,792)	3,040,101 (2,347,459–4,075,851)	2,361,431 (1,801,039–3,192,772)	1,708,950 (1,321,347–2,300,534)	1,440,229 (1,127,285–1,921,654)	− 47.7

In 2016, YLLs were highest among people aged 15–49 years (623,955, 95% UI; 442,103-888,510), followed by those aged 50–69 years (301,086, 95% UI; 216,478-428,083) (Additional file 1: Table S1). YLDs were highest in those aged 15–49 years (36,987, 95% UI; 22,578-55,926) and adults between 50 and 69 years (11,208, 95% UI; 6263-17,761]) (Additional file 1: Table S2). Between 1990 and 2016, DALYs and YLLs decreased in all age group over time, while there were variations in the YLDs across the age groups.

### TB mortality and DALYs attributable to individual risk factors

In Nigeria, alcohol use accounted for 13,196 (95% UI; 7277-20,605) TB deaths among HIV-negative people in 2016, followed by diabetes (1486 deaths [818–2493]) and smoking (942 deaths [349–1756]) (Additional file 1: Table S3). Proportionally, TB deaths that could be attributed to alcohol use was 38%, (95% UI; 23–52%), diabetes (4%, 95% UI; 3–6%) and smoking (3%, 95% UI; 1–5%) in 2016. The number of DALYs from TB due to alcohol use was 496,147 (95% UI; 283,342-777,331), followed by diabetes at 45,926 (95% UI; 26,297-75,452) and smoking at 32,369 (95% UI; 11,417-60,737) in 2016 (Additional file 1: Table S3).

## Discussion

In Nigeria, the prevalence of TB among HIV-negative people was 27%, the TB incidence rate was 158 per 100,000 population, and the total number of TB mortality was 39,933 in 2016. From 2000 to 2016, the age-standardised prevalence, incidence and mortality rates dropped considerably, with variations across the age groups. The number DALYs due to TB among HIV-negative Nigerians varied across the age groups; highest in those aged 15–49 years, followed by people aged 50–69 years and children under 5 years in 2016. Alcohol use accounted for the highest number of deaths and DALYs that could be attributed to TB in 2016, followed by diabetes and smoking, probably reflecting the high burden of TB among older adults.

Consistent with previous studies [1, 32, 33], this study showed that the prevalence and incidence of TB among HIV-negative people were higher in adults compared to children in Nigeria. Evidence has shown that not all individuals who are exposed to the *Mycobacterium tuberculosis* progress to having active TB infections. Studies from high burden TB environments suggest that approximately 20% of people maintain negative tuberculin skin tests throughout their lifespan despite repeated exposure to the mycobacteria [34]. In young children, active TB disease usually results from the haematogenous spread of the mycobacterium after primary infection, associated with subsequent pulmonary and extrapulmonary infections in some cases. In adults, however, TB infection is usually pulmonary and may reflect the reactivation of the latent TB infection (LTBI) from a primary site, which may partly be responsible for the increased prevalence and incidence observed in adults [35]. While only a limited number of individuals with LTBI progress to active TB disease, it is worth noting that one untreated infected person can transmit the disease to many healthy people, with broader implications for population health and TB control programmes [36, 37]. Early treatment of advanced LTBI in high TB-endemic countries like Nigeria is been advocated [38], and if the intervention is well implemented, it would reduce TB incidence and improve survival and productivity.

The present study showed that the number of deaths from TB mortality had dropped substantially over time in Nigeria, consistent with other reports [1, 2]. Similarly, between 2000 and 2016, this study indicated that TB incidence has declined. This improvement could be attributed to the scale-up of strategic policies and interventions, socioeconomic growth and a stable political environment [33, 39, 40], as well as increased developmental assistance for health and impact of the Millennium Development Goal agenda [17]. However, the WHO Tuberculosis Report 2017 indicated that TB incident cases have remained stagnant in Nigeria since the year 2000 [1]. The variation in the findings may be due to the data sources and methodological approach used wherein the WHO estimated TB incidence based on WHO notification [1]. The GBD study, however, employed a statistical triangulation method that utilised all data sources (including data from the WHO global TB database and surveillance data) in Nigeria for TB estimation [3, 41]. A recent systemic review conducted in Nigeria reported higher levels of MDR-TB compared to the WHO estimate [42]. Despite the differences in data sources and methodology, both the WHO and GBD study reported similar estimates for global TB incidence and mortality in 2016 [1, 28].

Globally, delayed TB diagnosis and treatment has been shown to increase the transmission of the mycobacterium, exacerbate the disease, increase the likelihood of mortality [43–45] and may be a reason for why TB incident cases have not reduced considerably compared to TB mortality [1, 3]. Evidence from regional areas of Nigeria found that delayed diagnosis and treatment of TB was due to factors such as a lack of awareness of TB symptoms by primary health professionals, older age, distance to the public health facility, male gender, and first clinic visit to a non-tuberculosis control programme providers [43, 46–48]. Additional studies have suggested that a lack of knowledge about TB in the community and patients preference for private health practitioners are the major reasons for why patients delay TB treatment [44, 45]. However, Lambert and Van der Stuyft argued that the failed health care system should be blamed and not the patient because there is limited evidence to indicate that health education about TB could reduce treatment delays [49]. Improving timely diagnosis and treatment of TB in Nigeria will require improved human resources, better coordination and decentralisation of TB control programmes [6], as well as increased and monitoring of public health financing [50].

The estimation of the population attributable risk for a specific disease or injury is crucial for health and other relevant agencies to identify opportunities for preventive efforts and policy priorities [22, 51, 52]. In the current study, we found that alcohol use, tobacco smoking and diabetes were essential contributors to the burden of TB in Nigeria. Studies have shown that the association between alcohol use [53, 54], smoking [55] and tuberculosis is due to impairment of the host immune system (innate and adaptive response), which increases vulnerability to TB infection, or reactivation of latent TB infection. Diabetes leads to increased susceptibility to tuberculosis through direct effects of hyperglycaemia and inadequate secretion of insulin at the cellular level, as well as indirect effects on specialised anti-TB immune cells (macrophages and lymphocytes), where chemotaxis, phagocytosis, activation and antigen presentation by macrophages are impaired [7, 56].

Evidence from regional areas of Nigeria has suggested that the lifetime prevalence of alcohol use was 57.9% [57], while the overall prevalence of current alcohol use ranged from 15 to 24% [57–60]. In Nigeria, there are some policy initiatives (excise tax on beer, wine and spirits, the national legal minimum age for on/off-premise sales of alcoholic beverages and regulations on alcohol advertising) to limit alcohol use. However, there is currently no written national action plan, nor is there a national monitoring system or enforcement of relevant policies to reduce alcohol use [61]. For tobacco smoking, an estimated 5.6% Nigerian adults aged over 15 years smoked tobacco products in 2017 [62]. Similar to alcohol use initiatives, strategic policies to support Nigerians to quit smoking [62], as well as efforts to prevent diabetes, are weak [63, 64]. Our finding implies that efforts must not only be made to strengthen the health system and its human resources for TB control but also calls for collaborative, targeted and measurable socioeconomic reforms that address issues of alcohol use, tobacco, smoking and vulnerabilities, galvanised with strong political support to reduce TB burden in Nigeria.

The current study has policy implications for national health agencies and development partners aiming to reduce the high burden of TB and improve the quality of life in Nigeria because it provides relevant country-specific epidemiologic data for TB disease. The World Health Organization End TB Strategy highlights priority areas for attention to end the global TB epidemic, with targets to reduce TB deaths by 95% and to cut incidence by 90% between 2015 and 2035 [65, 66]. The WHO domains include integrated, patient-centred TB care and prevention; bold policies and supportive systems; and intensified research and innovation. For Nigeria to achieve the WHO goal of ending TB, a multipronged approach will be needed. Those strategic measures will include closing the funding gaps for TB control programmes and reducing the reliance on international donors; scaling up the national immunisation schedule (including the anti-TB vaccine, bacillus calmette–guérin) in underserved areas; improving the political commitment at all levels of government; and strengthening the healthcare system and TB diagnosis and surveillance [3, 42], including improving coordination, integration and consistency in the primary health care structure through the National Primary Care Health Development Agency [6]. Additional measures to reduce the high burden of TB in Nigeria should also include initiatives to limit alcohol use and prevent tobacco smoking and diabetes [3].

The study has several methodological limitations, and they have been described in detail elsewhere [3, 21, 22]. Briefly, caution should be exercised when interpreting the study findings especially that vital registration and other high-quality data for TB are sparse at the subnational and national levels in Nigeria. Importantly, the availability of high-quality TB data at the subnational level is essential given differences in the socioeconomic and political situation in Nigeria which have been shown to influence healthcare and social policies [67, 68]. In the present study, the assessment of TB mortality was based on various modelling strategies of WHO notification and other published data. Consequently, the TB estimates for Nigeria with limited high-quality data are reflected in the wide uncertainty intervals. Efforts at improving both subnational and national research, surveys and vital statistics on TB disease burden are warranted in Nigeria to guide strategic policy interventions. While there is biological plausibility for the association between malnutrition and TB, the attributable burden of malnutrition due to TB was not examined in GBD 2016 because of limited evidence of a casual association. The limitations related to the estimation of TB incidence, YLLs, YLDs and DALYs using the GBD Bayesian meta-regression tool also applied to this study [24, 25]. Publication bias relating to the use of the GBD data may also be a limitation. Despite these limitations, this study provides comprehensive country-level epidemiologic data on TB disease burden and attributable risk factors to inform better TB prevention and control programmes in Nigeria, a country with Africa’s largest population of over 186 million people [40]. Future studies which investigate the rate of decline of TB incidence and mortality at the subnational level and whether those declines are fast enough to meet the WHO End TB Strategy may be warranted.

## Conclusion

Between 1990 and 2016, the present study showed a decreasing trend in TB disease burden among HIV-negative people in Nigeria. Despite this progress, TB disease remains a significant public health issue in the country. Efforts to ensure a further reduction in TB disease burden, as well as improve the health and well-being of Nigerians, will require a multipronged approach that includes increased funding and appropriate monitoring, health system strengthening and enhanced national and subnational surveillance for TB disease.

## Additional file

Additional file 1: **Table S1.** Number of years of life lost (with 95% uncertainty interval, UI) due to tuberculosis in Nigeria, 1990–2016. Table S2. Number of years of life lived with disability (with 95% uncertainty interval, UI) due to tuberculosis in Nigeria, 1990–2016. Table S3. Numbers of deaths and disability-adjusted life years (with 95% uncertainty interval, UI) from tuberculosis due to attributable risk factors in Nigeria, 1990–2016. (DOCX 25 kb)

## Abbreviations

CRA: Comparative risk assessment
DALYs: Disability-adjusted life years
GBD: Global burden of disease
LTBI: Latent tuberculosis infection
MDR-TB: Multidrug-resistant TB
TB: Tuberculosis
UI: Uncertainty intervals
WHO: World Health Organization
YLDs: Years lived with disability
YLLs: Years of life lost
