# Supplementary material for: Tuberculosis disease burden and attributable risk factors in Nigeria, 1990–2016
Source: Trop Med Health. 2018 Sep 25;46:34. doi: 10.1186/s41182-018-0114-9 (PMC6156953; doi:10.1186/s41182-018-0114-9)
Supplement: Supplementary file 1 — Table S1. Number of years of life lost (with 95% uncertainty interval, UI) due to tuberculosis in Nigeria, 1990–2016. Table S2. Number of years of life lived with disability (with 95% uncertainty interval, UI) due to tuberculosis in Nigeria, 1990–2016. Table S3. Numbers of deaths and disability-adjusted life years (with 95% uncertainty interval, UI) from tuberculosis due to attributable risk factors in Nigeria, 1990–2016. (DOCX 25 kb) [file 41182_2018_114_MOESM1_ESM.docx]

**Table S1: Number of years of life lost (with 95% uncertainty interval, UI) due to tuberculosis in Nigeria, 1990-2016**

| **Age** | **1990** | **1995** | **2000** | **2005** | **2010** | **2016** | **% change (1990-2016)** |
| --- | --- | --- | --- | --- | --- | --- | --- |
|  | **N (95%UI)** | **N (95%UI)** | **N (95%UI)** | **N (95%UI)** | **N (95%UI)** | **N (95%UI)** |  |
| Under 5 years | 791,339 (504,655-1,161,611) | 812,123 (521,502-1,218,921) | 728,830 (461,064-1,121,719) | 498,592 (317,695-743,811) | 401,131 (257,816-611,813) | 299,219 (1,82,705-487,268) | -62.2 |
| 5-14 years | 106,210 (73,882-148,413) | 116,415 (80,929-161,809) | 111,011 (75,603-151,187) | 85,381 (57,973-121,124) | 67,238 (46,333-93,493) | 57,280 (38,982-81,451) | -46.1 |
| 15-49 years | 1,111,631 (833,641-1,610,087) | 1,269,397 (890,035-1765,628) | 1,307,238 (881,549-1,928,331) | 1,050,575 (684,744-1,567,149) | 716,131 (483,951-1,040,178) | 623,955 (442,103-888,510) | -43.9 |
| 50-69 years | 530,314 (402,855-796,775) | 606,763 (436,549-894,067) | 628,844 (442,078-926,174) | 506,076 (358,341-747,094) | 350,457 (245,342-509,660) | 301,086 (216,478-428,083) | -43.2 |
| 70+ years | 159,885 (124,077-218,257 | 187,743 (145,706-252,374) | 192,390 (146,774-262,538) | 153,163 (115,681-204,028) | 108,468 (81,285-145,035) | 98,492 (74,786-131,535) | -38.4 |
| All Ages | 2,699,379 (2,164,767-3,566,684) | 2,992,440 (2,328,602-3,872,792) | 2,968,314 (2,268,571-3,985,376) | 2,293,787 (1,735,165-3,127,831) | 1,643,425 (1,253,758-2,230,092) | 1,380,032 (1,072,030-1,865,271) | -48.9 |

**Table S2: Number of years of life lived with disability (with 95% uncertainty interval, UI) due to tuberculosis in Nigeria, 1990-2016**

| **Age** | **1990** | **1995** | **2000** | **2005** | **2010** | **2016** | **% change (1990-2016)** |
| --- | --- | --- | --- | --- | --- | --- | --- |
|  | **N (95%UI)** | **N (95%UI)** | **N (95%UI)** | **N (95%UI)** | **N (95%UI)** | **N (95%UI)** |  |
| Under 5 years | 4,987 (2,518-8,668) | 5,971 (2,955-10,367) | 6,684 (3,393-11,744) | 6,147 (2,946-11,235) | 5,374 (2,451-9,730) | 4,409 (1,926-7,914) | -11.6 |
| 5-14 years | 3,241 (1,494-5,917) | 3,757 (1,801-6,602) | 4,510 (2,051-8,018) | 4,880 (2,059-8,819) | 4,549 (1,939-8,296) | 4,440 (1,825-8,328) | 37.0 |
| 15-49 years | 33,004 (20,187-48,441) | 39,500 (23,441-60,367) | 42,320 (24,944-64,556) | 39,200 (23,117-60,159) | 39,358 (23,866-59,579) | 36,987 (22,578-55,926) | 12.1 |
| 50-69 years | 10,883 (6,502-16,488) | 12,832 (7,505-20,026) | 14,466 (8,239-22,812) | 13,884 (7,785-21,786) | 13,174 (75,26-21,022) | 11,208 (6,263-17,761) | 3.0 |
| 70+ years | 3,051 (1,835-4,637) | 3,565 (2,167-5,572) | 3,807 (2,246-5,820) | 3,533 (2,119-5,526) | 30,70 (18,08-4,722) | 3,153 (1,875-4,958) | 3.4 |
| All Ages | 55,166 (35,369-77,402) | 65,625 (41,387-93,810) | 71,787 (45,698-102,852) | 67,644 (43,845-96,643) | 65,526 (41,396-94,495) | 60,197 (3,8620-86,097) | 9.1 |

**Table S3: Numbers of deaths and disability-adjusted life years (with 95% uncertainty interval, UI) from tuberculosis due to attributable risk factors in Nigeria, 1990-2016**

|  | **1990** | **1995** | **2000** | **2005** | **2010** | **2016** | **% change (1990-2016)** |
| --- | --- | --- | --- | --- | --- | --- | --- |
|  | **N (95%UI)** | **N (95%UI)** | **N (95%UI)** | **N (95%UI)** | **N (95%UI)** | **N (95%UI)** |  |
| **Deaths** |  |  |  |  |  |  |  |
| Tobacco smoking | 2,708 (1,318-4,655) | 3,006 (1,473-5,345) | 2,866 (1,356-5,242) | 1,837 (754-3,407) | 1,186 (441-2,271) | 942 (350-1,757) | -65.2% |
| Alcohol use | 23,960 (15,558-37,029) | 28,755 (18,430-43,433) | 30,663 (19,259-46,101) | 24,706 (15,513-38,053) | 16,641 (10,294-25,548) | 13,196 (7,278-20,606) | -44.9% |
| Diabetes | 2,223 (1,276-3,848) | 2,662 (1,479-4,585) | 2,846 (1,575-4,858) | 2,206 (1,192-3,793) | 1,571 (881-2,622) | 1,487 (819-2,493) | -33.1% |
| **DALYs** |  |  |  |  |  |  |  |
| Tobacco smoking | 90,853 (43,343-16,0847) | 100,817 (47,542-185,645) | 93,542 (42,121-176,480) | 61,123 (24,228-117,807) | 41,161 (15,304-79,565) | 32,369 (11,417-60,738) | -64.4% |
| Alcohol use | 883,113 (568,749-1,362,912) | 1,055,158 (668,473-1,576,994) | 1,131,214 (704,147-1,707,357) | 916,347 (577,158-1,440,611) | 623,823 (395,161-961,093) | 496,147 (283,343-777,332) | -43.8% |
| Diabetes | 68,537 (40,054-117,155) | 81,400 (47,063-133,665) | 87,003 (50,431-146,864) | 67,507 (38,642-114,130) | 48,554 (27,941-80,694) | 45,926 (26,298-75,453) | -33.0% |
